# Highly Aggressive Multiple Sclerosis Relapse During Pregnancy Following SARS-CoV-2 Infection: A Case Report and Literature Review

**DOI:** 10.7759/cureus.79718

**Published:** 2025-02-26

**Authors:** Roman Meyer, Patrick Sutak, Christian P Kamm, Lara Diem, Deepak Sharma

**Affiliations:** 1 Department of Critical Care Medicine, Lucerne Cantonal Hospital, Lucerne, CHE; 2 Department of Neurology, Lucerne Cantonal Hospital, Lucerne, CHE; 3 Department of Neurology, University Hospital Bern, Bern, CHE

**Keywords:** corona virus, ms (multiple sclerosis), post-infectious, sars-cov-2, tumefactive multiple sclerosis

## Abstract

We report a challenging case of a 32-year-old previously healthy pregnant woman at 17+2 weeks gestation with a new diagnosis of exceptional highly active relapsing-remitting multiple sclerosis (RRMS) triggered by a severe acute respiratory syndrome coronavirus 2 (SARS-CoV-2) infection. Remarkable clinical characteristics were the rapid clinical deterioration, the severity and the nature of the symptoms, including spastic tetraplegia, dyspnea, dysphagia, anarthria, and a severe pain syndrome, which resulted in the need for intensive care and mechanical ventilation within 24 hours. Relapse treatment, as well as symptomatic treatment, was challenging and complicated by pregnancy. Early diagnosis, consistent and persistent interdisciplinary management including six weeks stay in the intensive care unit and 3.5 months in neurorehabilitation, led to a full recovery of the patient and a healthy born child. In addition to the remarkable clinical characteristics, we report the challenging therapeutic measures throughout the hospitalization. This case report could, therefore, assist others who may be confronted with a similar situation.

## Introduction

Multiple sclerosis (MS) is a chronic inflammatory disease of the central nervous system (CNS) associated with demyelination and degeneration [1]. The course of the disease is highly variable; however, pregnancy usually improves the course of MS, especially during the last trimester [2]. There is evidence that severe acute respiratory syndrome coronavirus 2 (SARS-CoV-2) infections can trigger or cause various neurological disorders linked with the central and peripheral nervous systems. Possible mechanisms include cytokine-induced neuroinflammation, disruption of the blood-brain barrier, direct infection of glial cells, and autoimmune responses. Central nervous system diseases include demyelinating diseases such as acute disseminated encephalomyelitis (ADEM), acute necrotizing encephalopathy (ANE), and MS [3].

We report an unusual case of a young pregnant woman with a new diagnosis of exceptional highly active relapsing-remitting multiple sclerosis (RRMS) triggered by a SARS-CoV-2 infection. Within 24 hours of the onset of neurological symptoms, the patient required intensive care unit (ICU) treatment and had to be intubated.

Core symptoms included rapidly progressive spastic tetraparesis with eventually being tetraplegic, severe dyspnea, dysphagia, anarthria, bilateral visual impairment, and severe pain. We summarize the challenging and prolonged treatment of the patient due to the severity of the clinical presentation and the concurrent pregnancy, which ultimately resulted in a very positive outcome with almost full recovery. This case report could assist others who may be confronted with a similar situation.

## Case presentation

Clinical findings 

**Figure 1:**
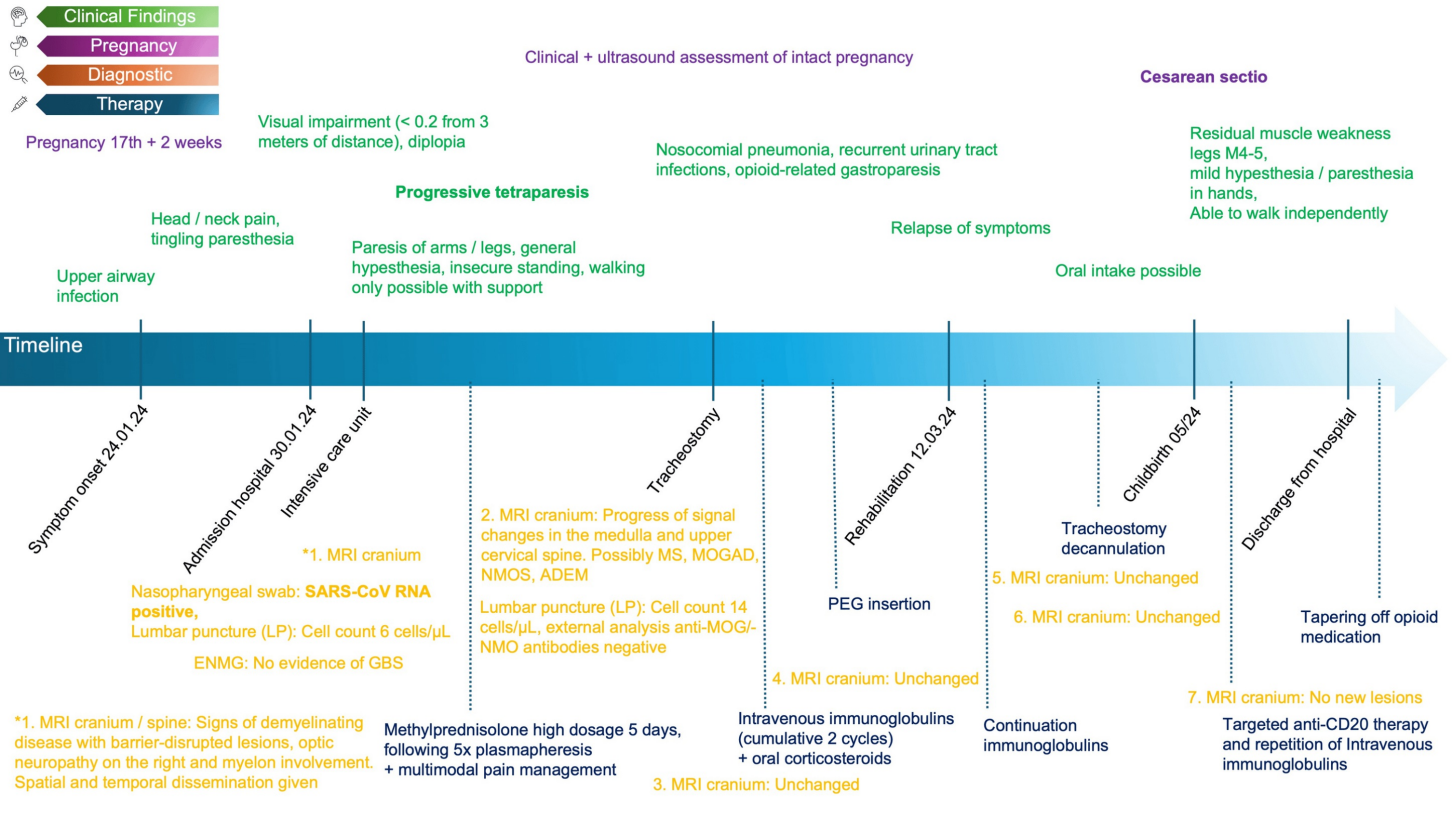
Case report timeline. Summary of clinical findings, diagnostic, and therapy. PEG: percutaneous endoscopic gastrostomy, MRI: magnetic resonance imaging, ENMG: electromyography, GBS: Guillain-Barré syndrome, MS: multiple sclerosis, MOGAD: Myelin oligodendrocyte glycoprotein antibody-associated disease, NMOS: neuromyelitis optica spectrum, ADEM: acute disseminated encephalomyelitis.

The timeline of the clinical course is also illustrated in Figure 1. Four days after the onset of an upper respiratory tract infection, later confirmed as SARS-CoV-2 infection, a 32-year-old previously healthy pregnant woman at 17+2 weeks gestation developed neurological symptoms including headaches, radiating neck pain, and a rapidly deteriorating spastic tetraparesis, which required admission to the emergency department. Symptoms worsened rapidly and drastically, and within just a few hours, the patient had tetraplegic with severe spasticity. The neurological examination revealed the following deficits: bilateral visual impairment (visual acuity < 0.2), dyspnea, anarthria, dysphagia, reduction in sensory functions, with a sensory truncal level advancing to C2 with severe hyperalgesia, increased reflexes of all extremities, spastic tetraplegia. Throughout the clinical course, cognitive function remained intact, enabling communication through eye closure and slight head nodding. Due to the clinical severity, the patient had to be directly transferred to the ICU with protective intubation on the same day.

Diagnostic assessment 

**Figure 2:**
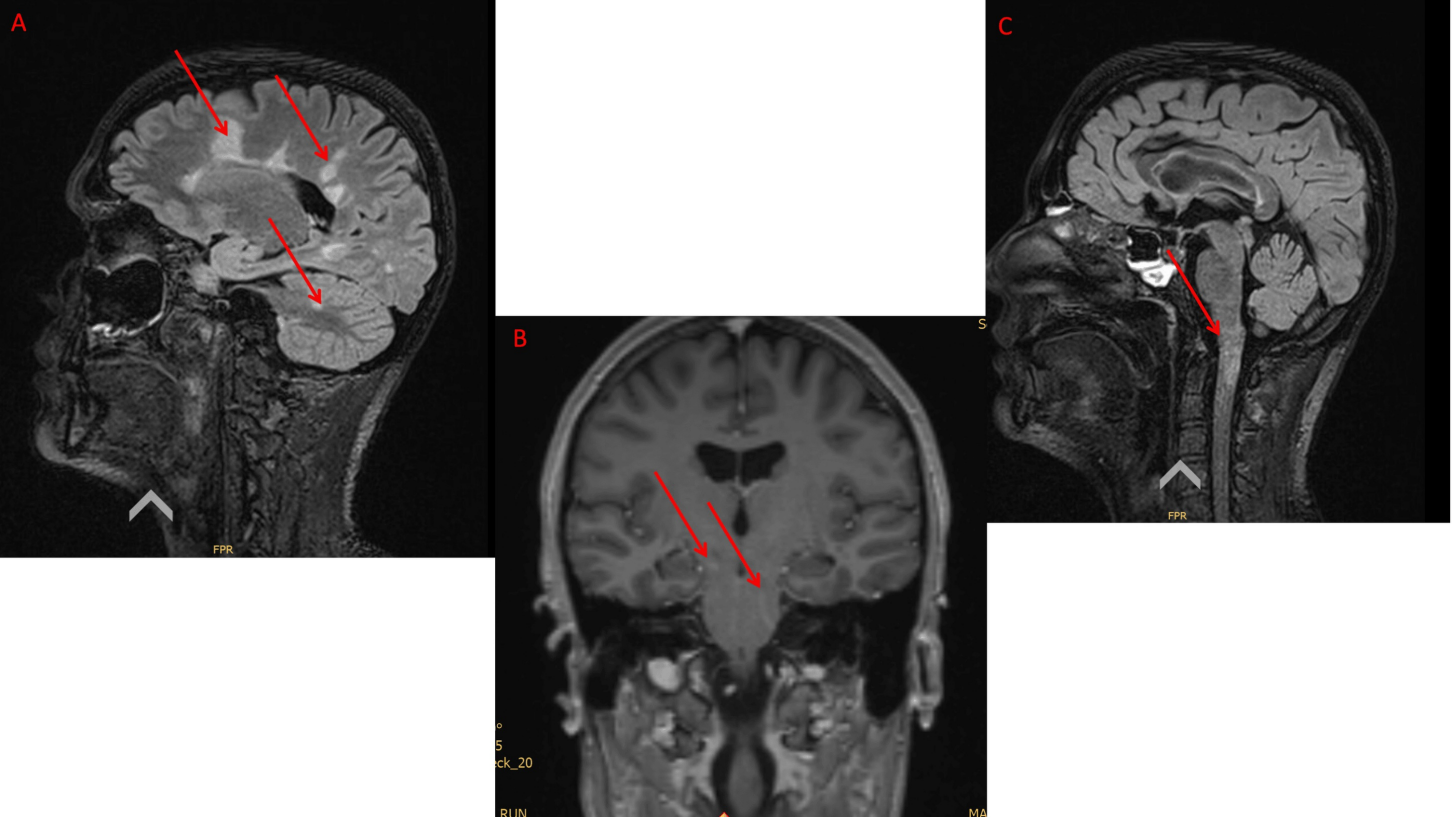
MRI cranium native and with contrast agent on admission day. (A) and (C) T2 flair sagittal: multifocal demyelination in the deep white matter in the supra- and infratentorial region, as well as in the pons and the cerebellar crus on the right. Demyelination is also found at the level of the medulla oblongata and at level C1 (red arrows). (B) T1 sagittal with gadolinium (red arrows).

The initial magnetic resonance imaging (MRI) of the brain and cervical spine revealed multiple demyelinating lesions with contrast enhancement in both the brain and spinal cord, as well as right-sided optic neuropathy (Figure 2). These findings fulfilled the McDonald 2017 criteria for RRMS. Cerebrospinal fluid (CSF) analysis revealed a mildly elevated cell count (6 cells/µl) without evidence of infectious agents and no indication of a blood-brain barrier disruption. However, positive oligoclonal bands (OCBs) were detected, suggesting intrathecal immunoglobulin synthesis. Testing for anti-neuromyelitis optica (NMO) and anti-myelin oligodendrocyte glycoprotein (MOG) antibodies in the serum in two reference laboratories (University Hospital of Zurich, University Hospital of Basel) with different assays was negative. Electroneurography (ENMG) showed no evidence of Guillain-Barré syndrome. As shown in Table 1, numerous additional laboratory tests revealed no evidence of any other rheumatological, autoimmune, or paraneoplastic diseases. Therefore, RRMS was finally diagnosed. 

**Table 1:** Laboratory findings. ANA: anti-nuclear antibodies, ANCA: anti-neutrophilic cytoplasmic autoantibody, TBE: tick-borne encephalitis, HTLV: human T-lymphotropic virus, MOG: myelin oligodendrocyte glycoprotein, MPO: myeloperoxidase, NMO: neuromyelitis optica, PR3: proteinase 3, RRMS: relapsing-remitting multiple sclerosis, QFN: Quantiferon.

Date	Test	Result
30.01.2024	Coronavirus SARS-CoV-2 RNA-nasopharyngeal swab	Positive
	Influenza A/B RNA-nasopharyngeal swab	Negative
31.01.2024	Anti-nuclear antibodies (HEp2 IIF)	1:320
	ANA pattern	AC-06
	ANCA	<1:40
	ANCA pattern	Negative
	PR3 antibodies sensitive	<0.1 IU/mL
	MPO antibodies sensitive	<0.1 IU/mL
	Hepatitis B HBs antibodies	Negative
	HTLV-1+2 Ab screening	Negative
	HTLV-1+2 Ab quantitative	Negative
	Varicella Zoster Virus IgG	671 mIU/ml
31.01.2024	Oligoclonal bands IgG, specific	>5
	Oligoclonal bands IgG, liquor vs. serum	Type 2
	IgG, liquor (intrathecal fraction, Reiber)	48%
	IgA, liquor (intrathecal fraction, Reiber)	53%
	IgM, liquor (intrathecal fraction, Reiber)	55%
	IgG-Index, liquor-serum	1.25
	Albumin-quotient, liquor/serum	3.6
31.01.2024 (External laboratory analysis)	TBE IgG/IgM	Negative
	West-Nil IgG/IgM	Negative
	Japanese encephalitis IgG/IgM	Negative
	Yellow fever IgG/IgM	Negative
	Dengue type 1-4 IgG/IgM	Negative
	*Mycoplasma pneumoniae* IgG/IgM	Negative
	*Borrelia burgdorferi* IgG/IgM	Negative
	*Treponema pallidum* Ab	Negative
	Anti-NMO (Aquaporin4)	Negative
	Anti-MOG	Negative
09.02.2024	Hepatitis C Ab	Negative
	HIV 1+2 Ab und HIV-1 p24 Ag	Negative
	Hep. B HBs Ab	<2 IU/L
	Cytomegalovirus IgG	<0.25 U/mL
	Cytomegalovirus IgM	Negative
25.04.2024	Hep. B–HBs Ab ql.	Negative
	Anti-GM1 IgG	<50
	Anti-GT1A IgG	<50
	Anti-GD1a IgG	<50
	Anti-GD1b IgG	<50
	Anti-GQ1b IgG	<50
	Anti-GM1 IgM	<50
	Anti-GT1A IgM	<50
	Anti-GD1a IgM	<50
	Anti-GD1b IgM	<50
	Anti-GQ1b IgM	<50
04.06.2024	QFN Mtbc-specific Ab	<0.35 IU/mL, negative
	QFN Mitogen (pos. control)	>10 IU/mL

Therapeutic intervention 

Due to severe dyspnea, intubation was necessary on the day of admission, and a tracheostomy was performed a few days later. Paxlovid® was not used due to the limited data and the possible teratogenicity of ritonavir and nirmatrelvir. 

Despite a five-day course of high-dose intravenous corticosteroid therapy (1000 mg methylprednisolone/day) and, subsequently, five sessions of plasmapheresis, the patient's condition worsened. Therefore, intravenous immunoglobulin therapy (1 g/kg body weight over three days) was administered. Concurrent oral corticosteroid therapy (1 g/kg body weight) was initiated. This led to a turning point, with a gradual slow improvement in symptoms. Due to the severe pain affecting the entire body and not responding to World Health Organization (WHO) class I and II treatments, a combination of a hydromorphone pump therapy (up to 18 mg/d) as continuous infusion and fentanyl pump therapy 30 mcg as reserve was given. Due to ongoing pain, transdermal fentanyl (up to 62 mcg/h), methadone (up to 60 mg/d), and hydromorphone 1.3 mg peroral was added in addition. However, co-analgesia with amitriptyline and lamotrigine was necessary to control the pain finally. Additionally, the patient found physical therapies, such as heat applications and gradual mobilization, to be very helpful. 

During ICU stay, mainly secondary to opioid-induced gastroparesis developed with progressive nausea and vomiting and a significant delay in the passage of liquid nutrition observed via nasogastric tube. Due to the mentioned dysphagia and gastroparesis, we started parenteral nutrition, which was replaced by a percutaneous endoscopic gastrostomy (PEG) tube a few days later. Treatment was complicated by existing pregnancy. All therapeutic decisions were made in consultation with gynecologists who closely cared for the mother and child. This included regular gynecological assessments and weekly sonographic surveillance of the child.

Outcome 

There was a slow continuous improvement of neurological deficits, as mentioned, requiring six weeks of ICU treatment, and mechanical ventilation was necessary for the overall five weeks. 

Initially, communication was restricted to eye closure and slight head nodding. As her condition improved, she was able to whisper individual words, though mild dyspnea on speaking persisted, without any reduction in oxygenation. The patient continued to experience burning pain throughout the body, primarily localized to the occipital and neck regions. This pain was largely consistent with central neuropathic pain, with musculoskeletal characteristics as well. Despite improvements in pain management with a multimodal analgesic and co-analgesic regimen, the patient occasionally reported pain spikes, reaching a level of nine on the numeric rating scale (NRS). Sensory symptoms included reduced tactile sensation symmetrically below C5, with significant pallhypesthesia pronounced in the legs. 

Additionally, the patient reported persistent weakness, particularly in the legs, and mild residual sensory disturbances in her hands. By the end of her ICU stay, spastic tetraparesis had significantly improved to a strength grade of M3-4. The patient was able to stand and walk a few steps with assistance, although she still experienced mild motor disturbances in both hands, which did not impede her ability to write. On neurological examination, the sensory level had shifted caudally, with reduced tactile sensation below C5. There was an improvement in respiratory function, with no signs of respiratory dysphonia or hypophonia. 

After ICU, the patient was transferred to our in-house neurorehabilitation clinic for 2.5 months. During this time, with intensive therapy, there was a steady improvement in all described sensorimotor symptoms. Her leg muscle strength had improved to M4-5, allowing her to walk independently without using walking aids. 

Nutrition was provided through the PEG tube for several weeks to assure adequate nutrition for the mother and child. Subsequently, a gradual reintroduction of oral feeding was possible. Upon admission to inpatient neurorehabilitation, the patient required concurrent administration of intravenous methadone, transdermal fentanyl, paracetamol, lamotrigine, and amitriptyline for pain management. An opioid rotation was implemented, transitioning from methadone through fentanyl to a hydromorphone patient-controlled analgesia (PCA) pump. However, a steady reduction in opioid requirements took several weeks. Nausea and gastroparesis also improved. Following delivery, we initiated and progressively increased the dose of pregabalin, which resulted in a significant reduction in pain. Amitriptyline was gradually tapered but remained part of the treatment regimen upon discharge. Lamotrigine was continued for the time being. By the end of the neurorehabilitation period, opioid therapy was no longer required.

During inpatient neurorehabilitation, immunotherapy was continued. Given the initial uncertainty regarding the diagnosis of MOGAD versus MS, we implemented the established intravenous immunoglobulin (IVIG) and corticosteroid regimen (with tapering) commonly used for MOGAD to ensure effective immunomodulation. Immunoglobulin therapy was maintained every three weeks at a dosage of 1 g/kg body weight over three days. Additionally, the patient received daily oral prednisolone at a dose of 40 mg. In total, glucocorticoids were administered for four months [4,5]. 

Regular gynecological assessments and sonographic evaluations of the child’s development were unremarkable throughout the pregnancy. An early cesarean section was initially planned at 34 weeks of gestation due to potentially harmful poly-medication. An emergency cesarean section was, however, necessitated two weeks earlier due to premature rupture of membranes (PROMs). The baby exhibited a positive progression during the monitoring period following delivery, showing no significant complications.

At the end of the inpatient neurorehabilitation, the patient exhibited residual muscle weakness in the legs with a strength grade of M4-5. She was able to walk independently without the use of walking aids. There were minor motor disturbances in both hands; writing was possible. Residual symptoms included mild hypesthesia and paresthesia in both hands. Nutritional intake was sufficient through oral means. There was no longer any respiratory dysphonia or hypophonia. Pain management was maintained with lamotrigine and pregabalin, while amitriptyline gradually tapered off, resulting in the absence of pain. Remission maintenance was ensured through continued intravenous immunoglobulin administration every three weeks.

In the outpatient setting, the anti-CD20 therapy with ocrelizumab was initiated and well tolerated by the patient. At the follow-up assessment in October 2024, the patient reported a stable clinical course without relapses or disease progression. She noted significant improvement in gait and balance but continued to experience mild intermittent paraesthesia in the upper extremities, particularly during physical activity, as well as persistent fatigue. Neurological examination revealed a normal gait, mild bilateral pallhypesthesia in the radial and malleolar regions, preserved muscle strength (M5/5) in all extremities, and mild dysmetria in the upper and lower extremities. MRI from September 2024 showed a stable lesion load without new or enhancing lesions. Given the stable clinical and radiological findings, IVIG therapy was extended to six-week intervals, with a planned further extension to eight-week intervals in February 2025 and discontinuation by June 2025. To manage persistent paraesthesia, an increase in pregabalin and a reduction in lamotrigine were considered. 

Patient perspective 

“When my entire body started tingling, I lost sensation, and my breathing became difficult, I was afraid that it would remain that way. During my stay at the neurological rehabilitation center, I gradually regained feeling throughout my body, bit by bit. The care provided by the doctors and nursing staff, the IVIG therapy, and especially the support from my family and friends helped me get through this difficult time. They constantly gave me strength and motivation to endure the rehabilitation process.” 

## Discussion

We report a challenging case of RRMS with an exceptional severe relapsing clinical disease onset formally leading to a formal expanded disability status scale (EDSS) of 9.5 and ICU requirement within one day. The initial relapse was probably triggered and/or amplified by a SARS-CoV-2 infection, and the management was complicated by pregnancy of the patient. Remarkable clinical characteristics were rapid clinical deterioration within 24 hours and the severity and nature of the symptoms.

In the most severe clinical manifestation, the patient presented with spastic tetraplegia, pronounced sensory disturbances, severe dyspnea and dysphagia, anarthria, and a pronounced therapy-refractory pain syndrome, which resulted in the need for intensive care and mechanical ventilation.

Many of these symptoms are not typical or common in RRMS, especially not in the early stages or during relapses. The most common symptoms in MS relapses are sensomotor deficits, fatigue, visual problems, walking difficulties, pain, and bladder problems; however, usually not in this severity. Severe dyspnea, dysphagia, and anarthria are uncommon symptoms of relapses and MS in general in the early stages [6]. This aggressive progression, as seen in our patient after an infection, would be possible for an ADEM. Ultimately, however, several clinical, radiological, and laboratory factors led us to the diagnosis of multiple sclerosis and not ADEM. The distinction between acute disseminated encephalomyelitis (ADEM) and multiple sclerosis (MS) is based on a combination of MRI findings, clinical presentation, and laboratory results. Clinically, encephalopathy is a common feature of ADEM but is rarely observed in MS. Our patient showed no signs of encephalopathy, making ADEM less likely. In terms of imaging, ADEM lesions are typically diffuse, poorly demarcated, and frequently involve the basal ganglia and thalamus, whereas MS is more commonly associated with periventricular lesions. In our case, the lesions were predominantly periventricular, with no involvement of the basal ganglia or thalamus, which is more characteristic of MS. Furthermore, cerebrospinal fluid (CSF) analysis supports the diagnosis, as oligoclonal bands (OCBs) are significantly more frequent in MS than in ADEM. A study found OCB in 84% of MS patients compared to only 20% of ADEM patients. Our patient tested positive for OCBs, further supporting the diagnosis of MS over ADEM [7,8]. 

Remarkably, cognition, including language understanding, was unaffected, which was important for the initial care of the patient, especially in the phase the patient was anarthric; however, they could communicate via “eye closer” and later “head nodding”. Such severe relapses, including the initial presentation, are more typical for NMO spectrum disorders (NMOSD) and MOG antibody diseases (MOGAD). Repetitive laboratory tests with different assays were, however, negative and the MRI was not typical in our opinion in this regard [9-11]. In addition to the rapid diagnosis, relapse therapy was necessary for relapse treatment, which included high-dose steroid therapy, plasmapheresis, and intravenous immunoglobulins, with steroids and immunoglobulins continued as maintenance therapy. The finally successful management of the severe pain required an extensive medication regimen with substances, including opioids. All described therapeutic measures, which were always carried out in consultation with the gynecologists, fortunately, did not result in any harm to the mother or child. Collaborative care across multiple specialties was pivotal, and this case underscores the need for research in optimizing evidence-based protocols to manage complex neurological cases.

It has been demonstrated in numerous studies that pregnancy has a beneficial effect on the relapse rate and severity of MS patients. Due to endocrine-modulating feedback, childbearing leads to the development of immune tolerance, which allows the growth of the semi-allogeneic fetal organism, expressing antigens inherited from the paternal side [12]. 

This leads to a less inflammatory immune system, with the above-stated positive consequences on relapses. In contrast to these facts, our patient experienced a severe relapse in the second trimester. In addition to the individual case reports, this rare finding was also observed in a small cohort of patients with radiologically isolated syndrome. It was found that pregnancy increases disease activity and even accelerates the first clinical presentation of the disease [13]. With regard to pregnancy, the fetus developed well at all times, as shown in regular sonography performed by obstetrics, and the child was finally delivered healthy by cesarean section. 

SARS-CoV-2 mainly affects the respiratory system, with common manifestations being fever, dyspnea, cough, and sore throat. In addition, SARS-CoV-2 infections are associated with various neurological diseases, including immune-mediated diseases such as acute encephalitis (AE) or ANE in the acute phase of the infection or ADEM, NMOSD or MOGAD in the subacute phase [14]. SARS-CoV-2 is believed to impact the nervous system through various direct and indirect mechanisms. These include direct damage to the CNS, peripheral nervous system (PNS), and muscle tissue, indirect vascular effects, para-infectious autoimmune responses such as cytokine storms, and post-infectious autoimmune responses involving cellular immunity and autoantibodies [15].

Given the described mechanisms, it has been proposed that SARS-CoV-2 could potentially trigger or exacerbate multiple sclerosis through various immune-mediated and inflammatory processes. The relationship between disease activity and COVID-19 remains, however, controversial. The occurrence of pseudo-exacerbations - transient clinical deteriorations triggered by an underlying SARS-CoV-2 infection - is considered an expected phenomenon. Indeed, there have been anecdotal reports of new demyelinating lesions or relapses following SARS-CoV-2 infection [16]. However, larger studies indicate that there is no increased risk of clinically and radiologically defined disease activity or worsening of motor and cognitive functions in the short- and long-term in patients with MS [17]. In Table 2, we summarize published MS cases after SARS-CoV-2 infections.

**Table 2:** Study cases of multiple sclerosis due to SARS-CoV-2.

Authors, year	Sex	Major manifestation	First symptoms after COVID-19 infection	Therapy	Outcome
Moore et al., 2021 [18]	Male	Blurry vision, vertigo	Two weeks	Methylprednisolone	Improvement vision and nystagmus
Palao et al., 2020 [19]	Female	Optic neuritis, anosmia	Three weeks	Methylprednisolone	Improvement visual acuity
Yavari et al., 2020 [20]	Female	Diplopia, paresthesia	Four weeks	Interferon beta	Improvement, persistent cough
Hu et al., 2023 [21]	Female	Blurry vision, numbness	Two weeks	Immunoglobulins, methylprednisolone	First improvement of vision, further no improvement (patient refused treatment)
Sarwar et al., 2021 [22]	Female	Blurry vision, paresthesia, and numbness	Three weeks	Remdesivir, dexamethasone	Improvement of vision and other neurological symptoms
Karsidag et al., 2021 [23]	Male/female	Numbness, weakness legs/jaw and facial pain/truncal ataxia, dysmetria	Four months/four weeks/two weeks	Methylprednisolone	Improvement
Pignolo et al., 2021 [24]	Male	Anosmia, paresthesia	One to two weeks	Corticosteroids, Interferon-beta	Improvement

While our patient exhibited comparable symptoms to those observed in the current cases, the rapid onset of neurological abnormalities, the severity and type of symptoms necessitating intensive medical intervention within six to seven days of SARS-CoV-2 infection, and the rapidity of the patient's deterioration are noteworthy. It is also noteworthy that the majority of cases described in the literature demonstrated improvement following the administration of high-dose corticosteroid therapy. In contrast, our patient exhibited an unusually aggressive disease course, particularly during the initial stages of hospitalization. In view of the available evidence, the hypothesis that SARS-CoV-2 infection contributed to both the onset of the disease and the severity of the symptoms is entirely plausible. However, it remains unclear to what extent a cytokine storm at the onset of the disease was responsible for the ICU liability.

## Conclusions

This case represents an exceptionally severe example of SARS-CoV-2-triggered RRMS in pregnancy with remarkable rapid deterioration and clinical characteristics requiring ICU management and mechanical ventilation within 24 hours and ICU treatment for six weeks. Relapse treatment was challenging, including high-dose corticosteroids, plasmapheresis, and immunoglobulins. Symptomatic therapy was challenging, especially regarding pain management and handling an anarthric intubated patient without cognitive deficits for six weeks.

Collaborative care across multiple specialties was pivotal for success, finally leading to a full recovery of the mother after an overall 3.5 months and a healthy child. Both mother and child recovered well following an emergency cesarean delivery, with the patient remaining stable under anti-CD20 therapy and IVIG.

## References

[1] Kamm CP, Uitdehaag BM, Polman CH (2014). Multiple sclerosis: current knowledge and future outlook. Eur Neurol.

[2] Varytė G, Zakarevičienė J, Ramašauskaitė D, Laužikienė D, Arlauskienė A (2020). Pregnancy and multiple sclerosis: an update on the disease modifying treatment strategy and a review of pregnancy's impact on disease activity. Medicina (Kaunas).

[3] Bhola S, Trisal J, Thakur V, Kaur P, Kulshrestha S, Bhatia SK, Kumar P (2022). Neurological toll of COVID-19. Neurol Sci.

[4] Bayry J, Lacroix-Desmazes S, Kaveri SV (2009). Novel therapeutic strategies for multiple sclerosis: potential of intravenous immunoglobulin. Nat Rev Drug Discov.

[5] Chen J, Huda S, Hacohen Y (2022). Association of maintenance intravenous immunoglobulin with prevention of relapse in adult myelin oligodendrocyte glycoprotein antibody-associated disease. JAMA Neurol.

[6] Nazareth TA, Rava AR, Polyakov JL, Banfe EN, Waltrip RW, Zerkowski KB, Herbert LB (2018). Relapse prevalence, symptoms, and health care engagement: patient insights from the Multiple Sclerosis in America 2017 survey. Mult Scler Relat Disord.

[7] Boesen MS, Blinkenberg M, Born AP (2020). Magnetic resonance imaging at baseline and follow-up to differentiate between pediatric monophasic acquired CNS demyelination and MS. Mult Scler Relat Disord.

[8] de Seze J, Debouverie M, Zephir H (2007). Acute fulminant demyelinating disease: a descriptive study of 60 patients. Arch Neurol.

[9] Cacciaguerra L, Flanagan EP (2024). Updates in NMOSD and MOGAD diagnosis and treatment: a tale of two central nervous system autoimmune inflammatory disorders. Neurol Clin.

[10] Jarius S, Aktas O, Ayzenberg I (2023). Update on the diagnosis and treatment of neuromyelits optica spectrum disorders (NMOSD) - revised recommendations of the Neuromyelitis Optica Study Group (NEMOS). Part I: Diagnosis and differential diagnosis. J Neurol.

[11] Sechi E, Cacciaguerra L, Chen JJ (2022). Myelin oligodendrocyte glycoprotein antibody-associated disease (MOGAD): a review of clinical and MRI features, diagnosis, and management. Front Neurol.

[12] Diem L, Hammer H, Hoepner R, Pistor M, Remlinger J, Salmen A (2022). Sex and gender differences in autoimmune demyelinating CNS disorders: multiple sclerosis (MS), neuromyelitis optica spectrum disorder (NMOSD) and myelin-oligodendrocyte-glycoprotein antibody associated disorder (MOGAD). Int Rev Neurobiol.

[13] Lebrun C, Le Page E, Kantarci O, Siva A, Pelletier D, Okuda DT (2012). Impact of pregnancy on conversion to clinically isolated syndrome in a radiologically isolated syndrome cohort. Mult Scler.

[14] Pilotto A, Masciocchi S, Volonghi I (2021). Severe acute respiratory syndrome coronavirus 2 (SARS-CoV-2) encephalitis is a cytokine release syndrome: evidences from cerebrospinal fluid analyses. Clin Infect Dis.

[15] Leven Y, Bösel J (2021). Neurological manifestations of COVID-19-an approach to categories of pathology. Neurol Res Pract.

[16] Michelena G, Casas M, Eizaguirre MB (2022). Can COVID-19 exacerbate multiple sclerosis symptoms? A case series analysis. Mult Scler Relat Disord.

[17] Prosperini L, Arrambide G, Celius EG (2024). COVID-19 and multiple sclerosis: challenges and lessons for patient care. Lancet Reg Health Eur.

[18] Moore L, Ghannam M, Manousakis G (2021). A first presentation of multiple sclerosis with concurrent COVID-19 infection. eNeurologicalSci.

[19] Palao M, Fernández-Díaz E, Gracia-Gil J, Romero-Sánchez CM, Díaz-Maroto I, Segura T (2020). Multiple sclerosis following SARS-CoV-2 infection. Mult Scler Relat Disord.

[20] Yavari F, Raji S, Moradi F, Saeidi M (2020). Demyelinating changes alike to multiple sclerosis: a case report of rare manifestations of COVID-19. Case Rep Neurol Med.

[21] Hu J, Huang L, Qiu Z, Liu Y, Shen K, Tang B, Qian J (2023). Case report: a novel case of COVID-19 triggered tumefactive demyelinating lesions in one multiple sclerosis patient. Front Neurosci.

[22] Sarwar S, Rogers S, Mohamed AS (2021). Multiple sclerosis following SARS-CoV-2 infection: a case report and literature review. Cureus.

[23] Karsidag S, Sahin S, Ates MF, Cinar N, Kendirli S (2021). Demyelinating disease of the central nervous system concurrent with COVID-19. Cureus.

[24] Pignolo A, Aprile M, Gagliardo C (2021). Clinical onset and multiple sclerosis relapse after SARS-CoV-2 infection. Neurol Int.

